# Management of adult renal trauma: a practice management guideline from the eastern association for the surgery of trauma

**DOI:** 10.1186/s12893-023-01914-x

**Published:** 2023-01-27

**Authors:** Hiba Abdel Aziz, Nikolay Bugaev, Gerard Baltazar, Zachary Brown, Krista Haines, Sameer Gupta, Lawrence Yeung, Joseph Posluszny, John Como, Jennifer Freeman, George Kasotakis

**Affiliations:** 1 Midwestern University, Drowners Grove, USA; 2 Tufts Medical Center, Boston, USA; 3 New York University Langone Medical Center, New York, USA; 4 United States Department of Army, Arlington County, USA; 5 Duke Medical Center, Durham, USA; 6 Nassau University Medical Center, East Meadow, USA; 7 University of Florida, Gainesville, USA; 8 Northwestern University, Evanston, USA; 9 Metrohealth Medical Center, Cleveland, USA; 10 Texas Christian University, Fort Worth, USA; 11 Duke University Health System, Durham, USA

**Keywords:** Kidney, Nephrectomy, Genitourinary tract, Injury, Wounds, Trauma, Blunt, Penetrating, Embolization, Renal laceration, Kidney preservation

## Abstract

**Background:**

The kidney is the most frequently injured component of the genitourinary system, accounting for 5% of all trauma cases. Several guidelines by different societies address the management of urological trauma. However, unanswered questions remain regarding optimal use of angioembolization in hemodynamically stable patients, indications for operative exploration of stable retroperitoneal hematomas and renal salvage techniques in the setting of hemodynamic instability, and imaging practices for patients undergoing non-operative management. We performed a systematic review, meta-analysis, and developed evidence-based recommendations to answer these questions in both blunt and penetrating renal trauma.

**Methods:**

The working group formulated four population, intervention, comparator, outcome (PICO) questions regarding the following topics: (1) angioembolization (AE) usage in hemodynamically stable patients with evidence of ongoing bleeding; (2) surgical approach to stable zone II hematomas (exploration vs. no exploration) in hemodynamically unstable patients and (3) surgical technique (nephrectomy vs. kidney preservation) for expanding zone II hematomas in hemodynamically unstable patients; (4) frequency of repeat imaging (routine or symptom based) in high-grade traumatic renal injuries. A systematic review and meta-analysis of currently available evidence was performed. RevMan 5 (Cochran Collaboration) and GRADEpro (Grade Working Group) software were used. Recommendations were voted on by working group members and concurrence was obtained for each final recommendation.

**Results:**

A total of 20 articles were identified and analyzed. Two prospective studies were encountered; the majority were retrospective, single-institution studies. Not all outcomes projected by PICO questions were reported in all studies. Meta-analysis was performed for all PICO questions except PICO 3 secondary to the discrepant patient populations included in those studies. PICO 1 had the greatest number of articles included in the meta-analysis with nine studies; yet, due to differences in study design, no critical outcomes emerged; similar differences among a smaller set of articles prevented observation of critical outcomes for PICO 4. Analyses of PICOs 2 and 3 favored a non-invasive or minimally invasive approach in-line with current international practice trends.

**Conclusion:**

In hemodynamically stable adult patients with clinical or radiographic evidence of ongoing bleeding, no recommendation could be made regarding the role of AE vs. observation. In hemodynamically unstable adult patients, we conditionally recommend no renal exploration vs. renal exploration in stable zone II hematomas. In hemodynamically unstable adult patients, we conditionally recommend kidney preserving techniques vs. nephrectomy in expanding zone II hematomas. No recommendation could be made for the optimal timing of repeat imaging in high grade renal injury.

*Level of evidence*: Guideline; systematic review, level III.

**Supplementary Information:**

The online version contains supplementary material available at 10.1186/s12893-023-01914-x.

## Introduction

The kidney is the most frequently injured component of the genitourinary system, and up to 5% of all trauma-related admissions involve the kidney [1–3]. As imaging modalities and endovascular technologies have advanced, non-operative management (NOM) approaches have become increasingly popular for high grade renal injuries. As a result, in the United States from 2002 to 2012, the rate of nephrectomy has decreased from 8.2% to 2.1% and 19.3% to 4.4% for blunt and penetrating renal trauma, respectively [4], and the current rate of operative intervention for high grade injuries is only 19% [5].

In the early 2000s, the Eastern Association for the Surgery of Trauma (EAST) developed practice management guidelines (PMGs) for the evaluation and management of genitourinary trauma [6, 7].

These guidelines did not use GRADE methodology and formulated the following recommendations:

- Conservative management of high-grade renal injury is feasible and can be supplemented by AE.
- Blunt trauma patients with major renal lacerations and a devascularized segment can be managed conservatively if clinically stable or operatively if there’s associated bowel or pancreatic injury.
- Penetrating renal lacerations can be managed non operatively in hemodynamically stable patients without associated injuries who have been staged completely with CT and/or intravenous pyelogram (IVP). If laparotomy is indicated for other injuries or if the injury is not completely staged prior to exploratory laparotomy then operative exploration is indicated.

In 2016, Bryk and Zhao built on these guidelines and published a “Guideline of guidelines” for urological trauma, noting evolving and differing recommendations among various medical organizations [8]. In 2019, the World Society of Emergency Surgery and the American Association for the Surgery of Trauma (WSES-AAST) offered broad options for kidney trauma management based on expert consensus with varying strengths of recommendations [9]. This was followed by a systematic review and meta-analysis from 2020 that reports in favor of minimally-invasive practices but acknowledges several unanswered questions [10].

Angioembolization (AE) has been increasingly used in hemodynamically (HD) stable patients with evidence of active extravasation, although questions remain on what specific subset of renal injury patients constitutes optimal candidates. The American Urological Association (AUA) recommends AE as an option in selected patients with bleeding from segmental renal vessels [11]. The European Association of Urology (EAU) recommends AE as an option for patients with active extravasation of contrast, arteriovenous fistula, pseudoaneurysm or a large perinephric hematoma [12]. WSES-AAST recommends AE if imaging demonstrates active bleeding, increased bleeding risk, or in the setting of non-self-limiting gross hematuria [9].

Previous guidelines provide conditional recommendation, for or recommend against exploration of stable retroperitoneal hematomas discovered on operation in HD unstable patients. WSES-AAST recommends exploration if the hematoma appears to be the sole cause of hemodynamic instability or is secondary to penetrating injury [9]. The EAU recommends against opening any stable retroperitoneal hematoma.

Attempts at renal salvage in the HD unstable patient may be more technically challenging and precarious compared to nephrectomy, and no recommendations currently exist for renorrhaphy or partial nephrectomy as an alternative to nephrectomy. Bryce and Zhao and the WSES-AAST note that outcomes for renovascular reconstructions are generally poor, often result in nephrectomy, and should only be performed in the setting of a solitary kidney or bilateral injury [8, 9].

Necessity, timing, and frequency of routine repeat computed tomography (CT) to assess stable patients undergoing NOM has not been standardized. The AUA recommends routine CT at 48 h for grade IV and V renal injuries; whereas, the EAU recommends repeat CT for grade V only. The Societe Internationale d’Urologie recommends CT for grades IV and V at 36–72 h if there is damage to the collecting system [8]. Petrone et al. recommend the use of repeat CT only on an individualized basis [10].

We aimed to address these four topics and developed evidence-based recommendations for the management of renal trauma patients through a systematic review and meta-analysis utilizing the Grading of Recommendations, Assessment, Development and Evaluation (GRADE) methodology [13, 14].

## Objectives

Our four population, intervention, comparator, and outcome (PICO) questions were defined as follows:

- PICO 1: In hemodynamically stable adult patients with renal trauma and evidence of active bleeding clinically or radiographically (P), should angioembolization (I), versus observation (C) be performed to decrease mortality, nephrectomy, or delayed hemorrhage necessitating intervention and the need for long term renal replacement therapy (RRT) (O)?
- PICO 2: In hemodynamically unstable adult patients with a stable zone II hematoma diagnosed intraoperatively (P), should renal exploration (I) versus no renal exploration (C) be performed to decrease mortality, nephrectomy, or delayed hemorrhage necessitating intervention, need for long term RRT, and angioembolization (O)?
- PICO 3: In hemodynamically unstable adult patients found to have an expanding zone II hematoma necessitating exploration (P) should total nephrectomy (I) versus renal preserving surgery (partial nephrectomy or primary repair) (C) be performed to decrease mortality, delayed hemorrhage necessitating intervention, or need for long term renal replacement therapy (RRT) and angioembolization (O)?
- PICO 4: In hemodynamically stable adult patients with high grade (AAST III-V) renal trauma managed non-operatively (P), should routine follow up CT abdomen (I) versus symptom-based CT abdomen (C) be performed to decrease delayed hemorrhage necessitating intervention (O)?

## Outcome measures

Pertinent outcomes were identified and discussed by the working group and voted by each team member on a scale from 1 to 9 per the GRADE methodology [13]. Critical outcomes were those with an average score of 7–9. Important outcomes received an average score of 4–6, and those with limited importance outcomes scored an average of 1–3. Only critical outcomes were included in our final PICO questions. Critical outcomes were mortality, delayed hemorrhage necessitating intervention, need for long term RRT, AE, and nephrectomy. Outcomes were matched to each PICO question and were considered collectively in developing recommendations. Please see Table 1 for the specific breakdown of scores for each PICO.

**Table 1:** PICO 1 to 4 outcomes rating

Outcome	PICO 1 Mean Score	PICO 2	PICO 3	PICO 4	Importance
Mortality	8.6	8.6	9		Critical
Delayed hemorrhage necessitating intervention	8.2	8.2	8	7.8	Critical
Long term RRT	7.5	7.2	7.3		Critical
Nephrectomy	8	7.8			Critical
Angio embolization		7.8	7.6		Critical

## Identification of references

Published literature was searched in May 2019 by a professional librarian utilizing MEDLINE (via PubMed), EMBASE (via Elsevier), Cochrane Central Register of Controlled Trials (via Wiley), Web of Science, and ClinicalTrials.gov databases. The following medical subject headings (MeSH) were included: kidney, nephrectomy, genitourinary tract, injury, wounds, trauma, blunt, penetrating, embolization, renal laceration, and kidney preservation in various iterations and combinations. Please see Additional file 1 for the full search strategy.

We limited our search to articles published in the English language. The date ranges for our literature search were 1969–2019, the workgroup elected to limit the literature search to 50 years.

Inclusion criteria were adult patients (> 15 years) with blunt/penetrating renal/renovascular trauma. Editorials, letters to the editor, case reports, commentaries, abstracts, reviews and animal studies were excluded. To be included in our final analysis, a clear comparison between intervention and comparator groups had to be present as well as at least one of the critical outcomes reported. Abstracts were screened independently by two work group members for inclusion in our meta-analysis. The studies were excluded based on one of the following criteria: wrong patient population, Wrong study design, Descriptive study, Wrong comparator, Wrong intervention, Wrong outcomes, not primary research, no intervention and comparison groups and old literature.

**Fig. 1:**
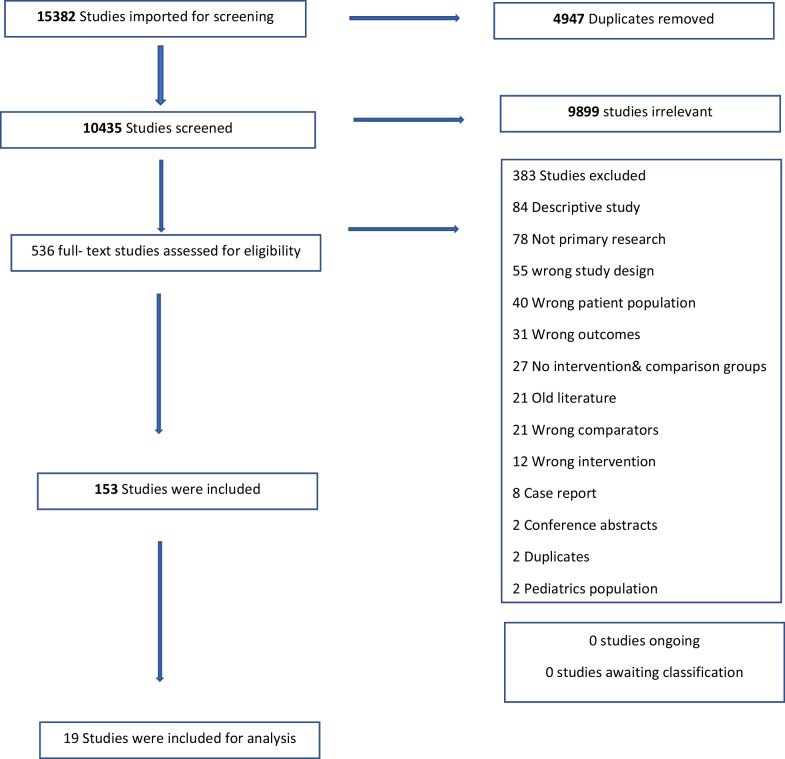
Prisma flow diagram for selected articles

Conflicts were adjudicated by a blinded third member. Full text review was performed in a similar fashion. References from included articles were reviewed for identification of potential additional articles. The PRISMA flow diagram for our systematic review is depicted in Fig. 1. Since the initial literature search concluded in 2019, the first author performed an updated literature search to identify any recently published relevant articles. None was identified.

## Data extraction and methodology

Data extraction was performed from each included study using standardized data collection sheets. Each study had two reviewers extracting data with differences being reviewed by a third reviewer. Data extracted included: reference first author/publication year, study title, study design, population, intervention, comparator, overall study size, intervention group size, comparator group size and outcomes in intervention vs comparator groups.

Meta-analysis was performed in Review Manager (RevMan, 5.3. Copenhagen: The Nordic Cochrane Centre, The Cochrane Collaboration, 2014) with random-effects modeling to generate forest plots. Treatment effects were calculated with each study weight being proportional to the number of subjects it contributed to each outcome. For dichotomous data points (mortality, nephrectomy, delayed hemorrhage necessitating intervention, need for long term renal replacement therapy and angioembolization), odds ratios were calculated for the intervention versus the comparator groups. Heterogeneity was calculated and quantified with I^2^. Low degree of heterogeneity had I^2^ values less than 50%, those with moderate heterogeneity had I^2^ values of 50–74%, and ones with I^2^ values greater than 75% were indicative of high heterogeneity [15]. The GRADE framework was applied to all quantified outcomes for assessment of bias, publication bias, inconsistency, imprecision, and indirectness. Evidence profiles were created for each PICO using GRADEpro GDT software (GRADEpro Guideline Development Tool. McMaster University, 2015). All workgroup members voted independently to reach consensus on the proposed recommendations.

## Results

A. PICO 1: In HD stable adult patients with renal trauma and evidence of active bleeding clinically or radiographically (P), should AE (I), versus observation (C) be performed to decrease mortality, nephrectomy, delayed hemorrhage necessitating intervention and the need for long term RRT (O)?

### Qualitative synthesis

There were a total of nine retrospective studies which addressed HD stable patients. Six studies directly compared AE to observation. [16, 18–20, 22, 23] In three studies, AE and observation were both included in the conservative management arm and compared to operative intervention [17, 21, 24]. Significant selection bias existed among all the studies given the retrospective study designs. McPhee et al. assessed both blunt and penetrating renal injuries [22]. Other studies examined only blunt injuries [16, 19, 23]. Five studies chose to focus only on blunt high grade (III–V) renal trauma (HGRT) [17, 18, 20, 21, 24].

Varying approaches to diagnostic imaging confounded comparison of the studies [16–24]. Renal injuries were classified retrospectively according to AAST renal injury scale by a single trauma surgeon [16, 18], a trauma surgeon and a radiologist [17], a single radiologist [21, 22, 24], two radiologists [20] or not mentioned [19, 23]. Initial abdominal CT scans with IV contrast were used for renal injury grading in all studies [16–24]. Follow up imaging to assess evolution of reported injuries in patients managed conservatively was not obtained [17, 18, 20, 22–24], was obtained in some patients only [16] or in all patients [20] using US or CT either routinely [21] to detect early complications or periodically until resolution of hematoma [19].

A protocol [17, 19] or predetermined pathway/algorithm for management of renal injury patients was utilized in a few studies [17, 19–21]. Hence, the trigger for initiating embolization in patients failing observation was not always evident and ranged from radiographic evidence of ongoing bleeding (pseudo aneurysm (PSA) or contrast extravasation (CE)) to ongoing unspecified transfusion requirements [16]. Charbit et al. performed angiography on all patients with HGRT who needed to be transfused two or more units of PRBCs without other causes of active hemorrhage and had the following associated CT parameters of renal bleeding: intravascular CE and a large perirenal hematoma [17]. Chow et al. performed angiography in HD stable patients with documented renal arterial extravasation on CT if immediate surgical exploration was not required for associated injuries and embolization was performed in those patients with evidence of contrast extravasation on angiography; otherwise, observation was initiated [18]. In the Hagiwara study, HD stable patients with or without fluid resuscitation underwent CT, and if emergency surgery was not required: Grade I–II were observed while grade III–V underwent angiography within three hours of initial CT [19]. AE was performed for extravasation of contrast medium from renal artery or presence of an arteriovenous fistula (AVF). In the study by Lin et al., angiography was indicated if CE from the kidney was found on CT or if there was clinical suspicion of persistent renal bleeding (four or more units of blood daily after excluding other sources) [20]. AE was performed on those patients with positive angiographic findings including renal CE, PSA and AVF. McGuire et al. referred HD stable patients with ongoing blood loss for AE [21]. Reason for AE was not clear in two papers [22, 24]. In the study by McPhee et al., three patients all with penetrating trauma underwent AE [22]. For Menaker et al., routine angiography was not performed for blunt renal injury regardless of the grade and angiography was performed for specific CT findings (active CE, other solid organ injuries requiring angiography, and HGRT injuries) or at the discretion of the trauma surgeon [23].

While not clearly mentioned in each study [18, 20–22], maintenance of HD stability varied across the studies with patients receiving undefined packed red blood cell (PRBC) transfusions [16, 23, 24], >/= two PRBCS per protocol [17] or fluid resuscitated even if initially HD unstable provided they could be stabilized by fluid resuscitation. [18].

Injury severity score (ISS) and AAST grades between the observation and the intervention arms were either not compared [18, 19, 22] or were not statistically significant [16, 17]. McGuire et al. found that those who required immediate treatment had a significantly higher proportion of grade V injuries than those treated expectantly (65% vs 14.4%, p < 0.001), but ISS did not attain statistical significance [21]. Menaker et al. noted that patients selected for angiography had a significantly higher percentage of high-grade renal injuries (III–V) than those managed with observation alone (p < 0.001) [23].

### Quantitative synthesis

**Fig. 2:**
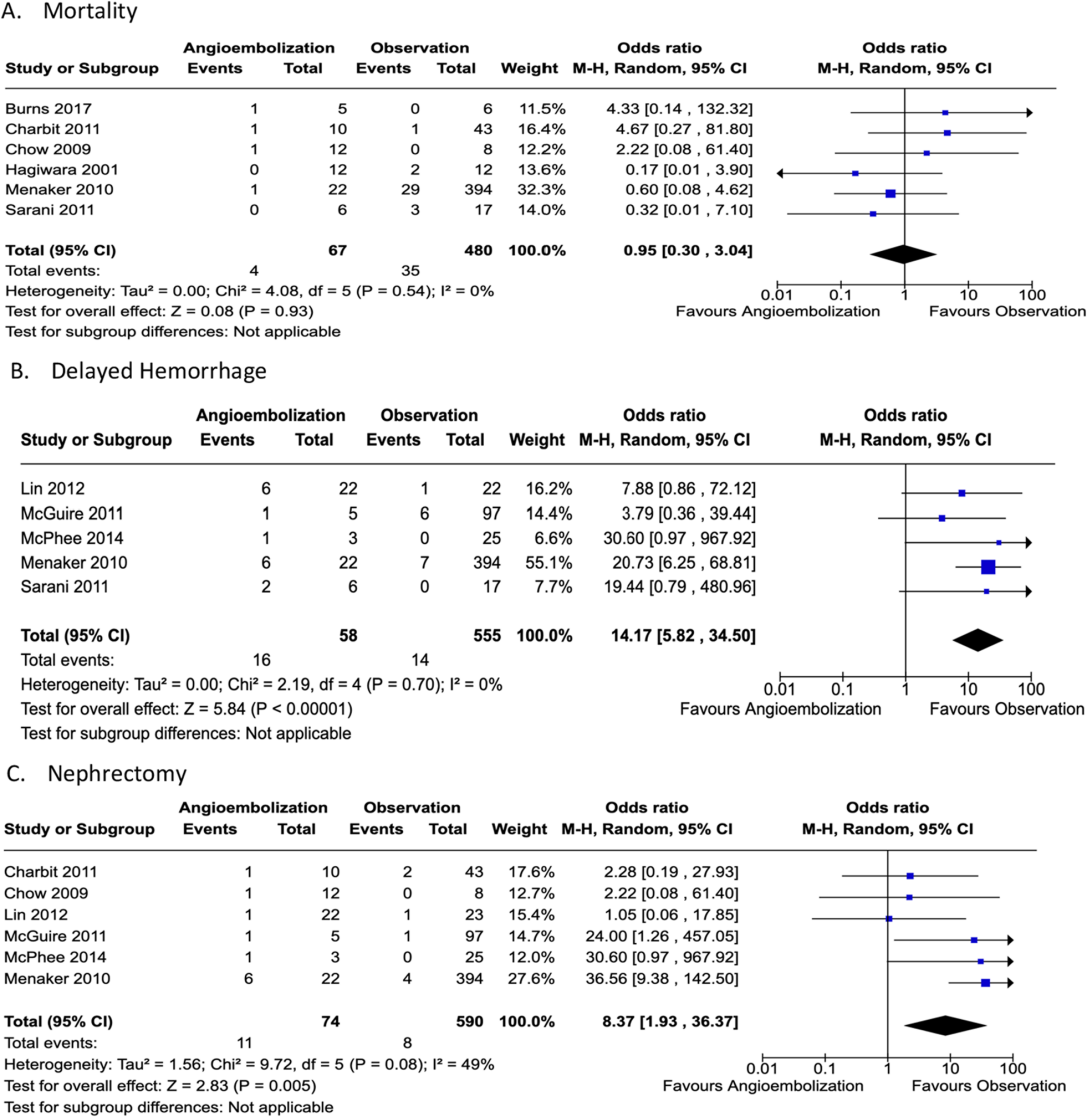
Forest plots illustrating outcomes for PICO 1: AE vs. observation

Across all the analyzed studies, a total of 116 patients were included in the AE group while 603 patients underwent observation. Among the intended outcomes, need for long term RRT was not investigated in any of the studies. Across the included studies, mortality ranged from 0% [19–22, 24] to 17% [16] in the AE group and from 0% [16, 18, 20–22] to 17.6% [19] in the observation group with a heterogeneity of 0% (p = 0.564). The total deaths in the former group was 1 versus 34 in the latter (see Fig. 2A).

Five articles [20–24] noted the incidence of delayed hemorrhage necessitating intervention in AE cohort was 20% [21] to 33.3% [22, 24]; 16 patients total in contrast to 0% [24] to 6.2% [21] in the observation cohort; 15 patients with all five studies favoring the observation arm. Heterogeneity was 0% (p = 0.709) (Fig. 2B). Nephrectomy was discussed in seven studies and varied in the observation group from 0% [18, 22, 24] to 10% [17], a total of 9 patients compared to 0% [24] to 33.3% [22], six total patients in the AE group with five articles [17, 18, 21–23] favoring observation. Heterogeneity was 48.6% (p = 0.084) (see Fig. 2C).

### Grading the evidence

**Fig. 3:**
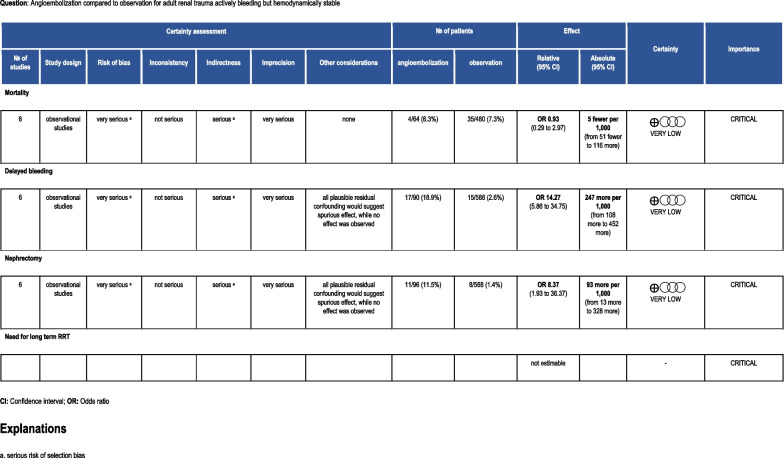
PICO 1 evidence profile

The individual studies suffered from a serious risk of bias, indirectness, inconsistency and imprecision of the data (Fig. 3). The retrospective, single-center design that prevailed among the studies can also promote selection bias. Polysystem trauma may have precluded a correlation analysis between the hemodynamic status or PRBC transfusion requirements and the need for AE of the kidney. Indications for angiography varied between studies. In addition, transfusion requirements prior to embolization varied between studies suggesting that some patients may have been embolized unnecessarily.

### PICO 1 recommendation

The majority (7) of the workgroup members rendered a No recommendation vote. Hence in HD stable adult patients with renal trauma and evidence of active bleeding clinically or radiographically (P), no recommendation can be made for or against angioembolization (I), versus observation (C) to decrease mortality, decrease need for long-term RRT, delayed hemorrhage necessitating intervention and nephrectomy (O).

B. PICO 2: In HD unstable patients with a stable zone II hematoma diagnosed intraoperatively (P), should renal exploration (I) versus no renal exploration (C) be performed to decrease mortality, nephrectomy, delayed hemorrhage necessitating intervention, need for long term RRT and angioembolization (O)?

### Qualitative synthesis

A total of three studies, two retrospective [25, 26] and one prospective [27] were included. The latter solely addressed blunt renal trauma while the former studies focused on penetrating injuries only.

Rostas and colleagues opted to selectively explore renal injuries based on patient stability and intraoperative findings with the ultimate decision for renal exploration made by the operating surgeon. Hemodynamic instability was defined as preoperative or intraoperative SBP less than 90 mmHg [26]. Explored renal injuries were either graded intraoperatively or by CT. Despite a statistically significant higher mean grade renal injury in the perirenal hematoma exploration cohort, both groups had comparable ISS and other abdominal organs injured. In their study, patients in whom Gerota’s fascia was not explored were felt to be HD unstable secondary to bleeding sources other than the kidney.

Costa et al. evaluated the management of forty-one patients with type II penetrating renal hematomas [25]. However, in twenty-six patients, the decision to explore the renal hematoma was not always clear. Eight of those patients had sustained gunshot wounds, but none of the patients had an expanding hematoma or other stigmata of continued hemorrhage.

Although in Toutouzas study the intended outcome was not evaluating Gerota’s exploration, his series investigated blunt HGRT [27].

### Quantitative synthesis

**Fig. 4:**
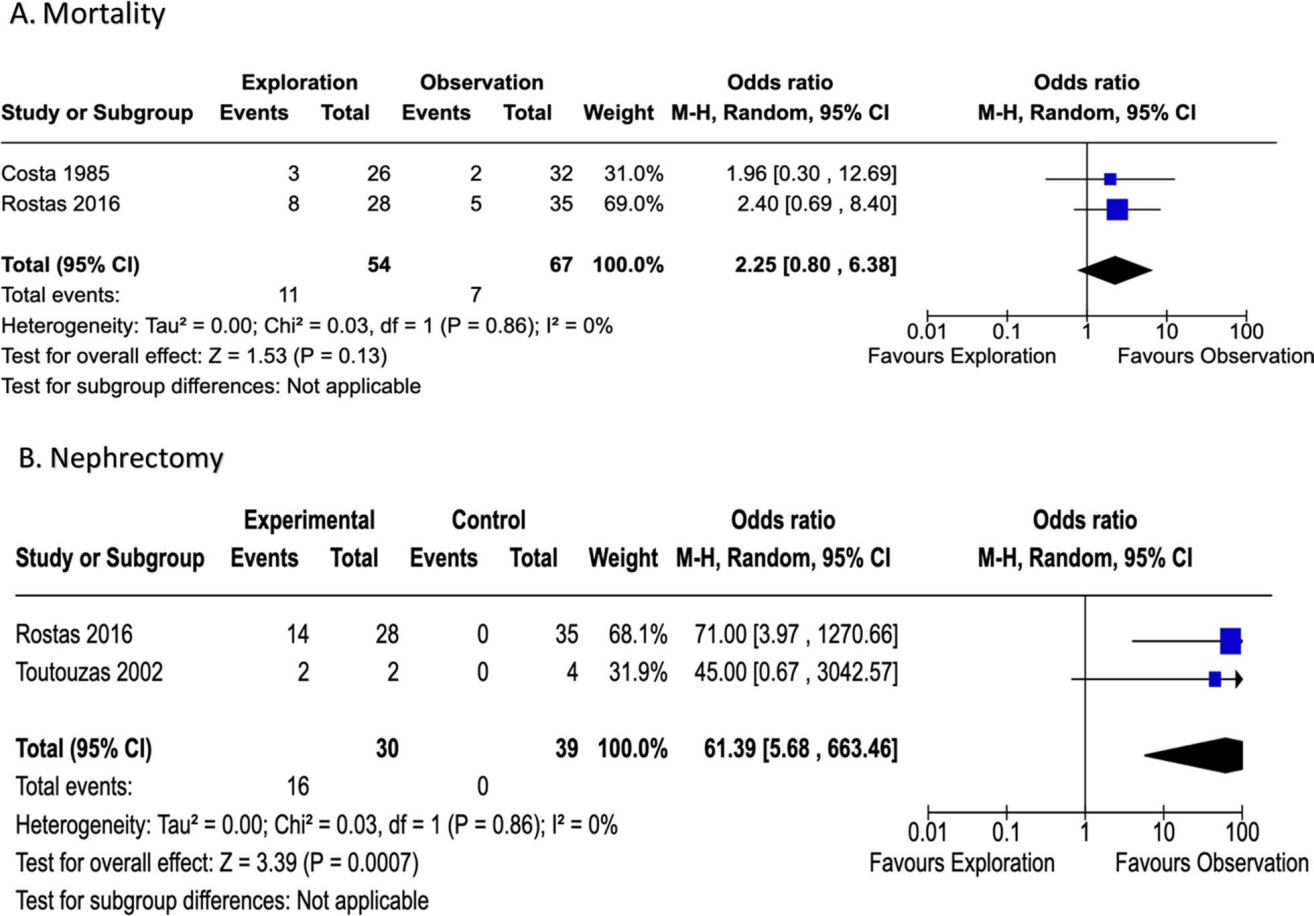
Forest plots illustrating outcomes for PICO 2. Renal exploration vs. no renal exploration

One hundred seventy-three patients were included in all studies. Seventy-four patients with renal hematoma underwent Gerota’s exploration, the remaining patients had stable hematomas which did not necessitate exploration. The critical outcomes of mortality and nephrectomy were each discussed in two studies (Fig. 4). Eight (29%) mortalities occurred in the former group and five (14%) in the latter, with a heterogeneity of 0% (p = 0.859). These findings favored observation.

Both Rostas and Toutouzas investigated the outcome of nephrectomy. Findings from both studies favored the observation arm with a heterogeneity of 0% (p = 0.855) [26, 27]. None of the studies addressed the outcomes of delayed hemorrhage necessitating intervention, AE or need for long term RRT.

### Grading the evidence

**Fig. 5:**
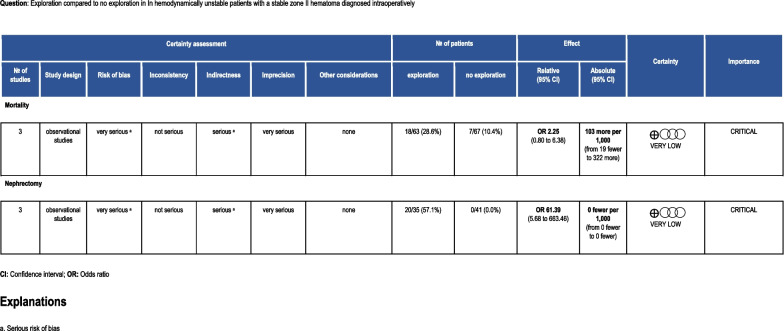
PICO 2 evidence profile

Overall, the quality of evidence for PICO 2 is very low due to publication bias, imprecision, indirectness, and inconsistency (Fig. 5). This is compounded by the small sample size in each study which can lead to sampling error. After presenting this work at The EAST trauma guidelines committee and polling trauma surgeons in attendance, the EAST adult renal trauma workgroup reached a consensus.

### PICO 2 recommendation

Six workgroup members rendered a “conditionally recommend against” renal exploration vote. In HD unstable patients with a stable zone II hematoma diagnosed intraoperatively (P), we conditionally recommend against renal exploration (I) vs no renal exploration (C) to decrease the incidence of mortality and nephrectomy (O).

C. PICO 3: In HD unstable patients found to have an expanding zone II hematoma necessitating exploration (P) should total nephrectomy (I) versus attempted kidney preserving surgery (partial nephrectomy or primary repair) (C) be performed to decrease mortality, delayed hemorrhage necessitating intervention, or need for long term RRT and AE (O)?

### Qualitative synthesis

One prospective cohort and four retrospective studies with data relevant to this PICO question were identified [28–32]. One study was specifically designed to compare the two surgical approaches with 1:1 patient matching. Both blunt and penetrating renal trauma were included, with the former accounting for 1/3 of the total population.

Brown attempted vascular repair for isolated renovascular injuries with a calculated 59.3% incidence of successful repair [29]. The authors observed an association between the extent and complexity of the revascularization technique and the occurrence of kidney dysfunction. In their series, immediate nephrectomy was performed for control of hemorrhage, irreparable damage, or expediency due to patient’s overall condition and associated injuries. Kuo noted renal injury grade, ISS, and transfusion requirements to be significant contributing factors in nephrectomy patients [30]. 89% nephrectomy patients had grade IV-V injuries with 62.5% of them presenting in shock. The previous risk factors were verified in a prospective study by Nicol who found a statistically significant difference in renal injury score, trauma score, blood transfusion and shock status at presentation between the nephrectomy and the renal salvage cohorts [28].

Velmahos assessed the impact of nephrectomy in postoperative renal failure between nephrectomy and nephrorrhaphy groups matched 1:1 with regard to injury severity and organs injured [31].

Volezke reported their experience with the management of renal gunshot wounds (RGSW). In their study, the outcomes of mortality, delayed hemorrhage necessitating intervention and AE were explored [32].

### Quantitative synthesis

Brown et al. included a total of 154 patients; 105 underwent nephrectomy, and 49 underwent attempted vascular repair for isolated renovascular injuries with a calculated 59.3% incidence of successful repair. Four (8%) of 49 patients subsequently underwent nephrectomy secondary to delayed hemorrhage [29].

In the study by Kuo, ten patients were included. The nephrectomy cohort suffered 22% mortality risk, and no mortality was reported in the renal preservation cohort [30]. Nicol compared mortality between 13 nephrectomy patients and 22 renal preservation patients [28]. For these authors, renal salvage rate was 73.5% with a 100% incidence of survival compared to a 23% reported mortality in the nephrectomy arm.

Velmahos assessed renal dysfunction as an outcome and ascertained its occurrence in 8 (14%) of 59 nephrorrhaphy patients versus 6 (10%) of 59 nephrectomy patients (p = 0.57) with one post nephrectomy patient remaining hemodialysis dependent 19 months after injury [31].

Volezke compared 30 nephrectomy to 105 kidney repair patients and reported an overall renal salvage rate of 85.4% after RGSW with one (1%) of 100 patients in the kidney preservation group requiring AE and two (2%) of 104 patients progressing to nephrectomy [32]. Overall survival rate was 90.6% with 2.9% (3 of 105 patients) mortality in the kidney preservation group in contrast to 33% mortality (10 of 30 patients) in the nephrectomy group.

For this PICO question, we were unable to perform meta-analysis/Forest plots as all studies except one [28] skew towards more heavily injured patients in the nephrectomy group.

### Grading the evidence

**Fig. 6:**
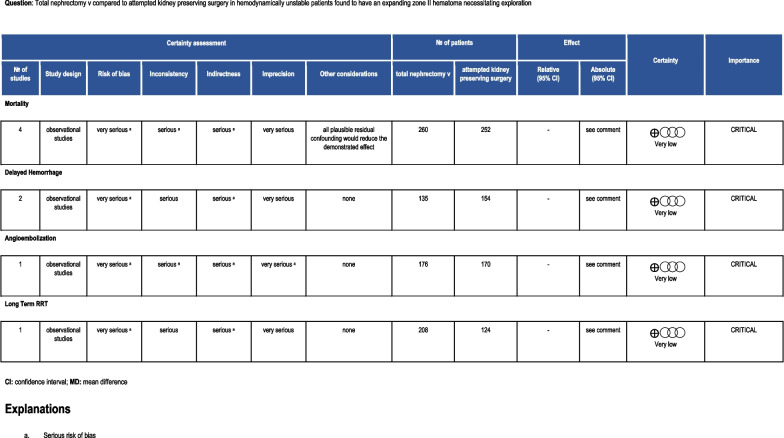
PICO 3 evidence profile

We rated the overall certainty in the evidence of effects as very low based on the lowest certainty in the evidence for the critical outcomes, and downgrading for study limitations, imprecision and indirectness (Fig. 6). While the need for nephrectomy may be a harbinger of the overall severity of the patient’s condition and injury grade, it may be argued that the need for operation on the kidney may be the cause for renal dysfunction or just an indicator of severe injury that ultimately leads to organ compromise. Nevertheless, preserving the kidney may aid in obviating the need for future dialysis. The panel judged, based on the available very low certainty evidence, that the expected net benefit favored kidney preservation. While it is a cogent argument for the trauma surgeon to attempt renal preserving surgery given these desirable outcomes, considerations of surgical expertise, feasibility, and the timely completion of the operation to avoid added physiological compromise in a critically injured patient always takes precedence.

### PICO 3 recommendation

Six workgroup members provided a “conditionally recommend against” total nephrectomy vote. Hence in HD unstable patients found to have an expanding zone II hematoma necessitating exploration (P), we conditionally recommend against total nephrectomy (I) versus attempted kidney preserving surgery (partial nephrectomy or repair) (C) to decrease mortality, delayed hemorrhage necessitating intervention, AE, and need for long term RRT (O).

D. PICO 4: In HD stable adult patients with high grade (AAST III-V) renal injuries managed non-operatively (P), should routine follow up CT abdomen (I) versus symptom-based CT abdomen (C) be performed to decrease delayed hemorrhage necessitating intervention (O)?

### Qualitative synthesis

Two studies compared the utility of routine follow up CT abdomen versus symptoms-based imaging in HD stable patients with HGRT undergoing conservative management [33, 34]. Both were retrospective. One excluded patients who underwent initial treatment with either embolization or nephrectomy, the other did not [33, 34].

Details of initial and follow up imaging were either retrospectively reviewed by a uroradiologist [34] or retrospectively gathered from the medical records [33]. The AAST organ injury severity scale was used to grade the initial injury on imaging. Davis et al. [34] repeated imaging for sepsis, hemodynamic instability, decreased hemoglobin, and persistent hematoma. Polytrauma patients undergoing further imaging for non-renal injury but with urinary tract assessment available were included in the routine imaging group. Routine imaging was done after 48 h (range 4–240), at a meantime of 35.9 days, and symptom-based imaging was performed at 17.1 days (range 3–45); CT was the preferred modality in > 80% in both cohorts and grade III–V, comprising 63% of both groups.

Re-imaging indications for Aldiwani were fever, pain or suspicion of active bleeding [33]. Most of the renal trauma resulted from blunt mechanisms (74–97%), and grade III-V comprised 58–64% of the injuries. Whereas, Davis et al. performed both inpatient and outpatient repeat imaging [34], Aldiwani et al. only re-imaged inpatients [33]. Early inpatient re-imaging was executed in 59 of 90 patients. Mean time from initial to repeat CT scan was 3.4 days. The majority were planned re-evaluation scans in 83% of patients, and the remainder were secondary to clinical indications.

### Quantitative synthesis

**Fig. 7:**
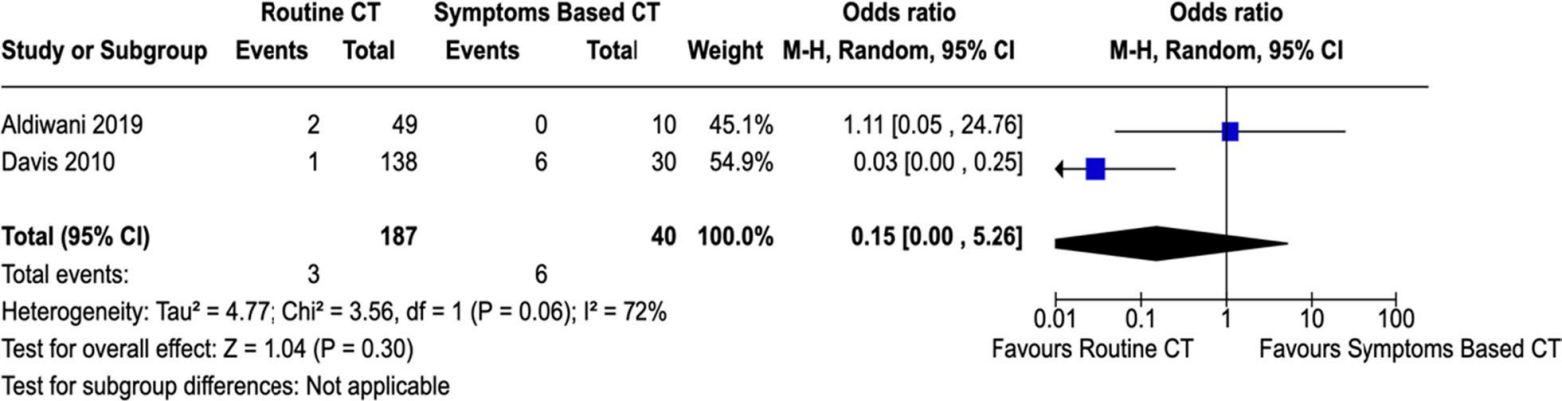
Forest plots illustrating outcomes for PICO 4 (routine imaging vs. symptoms based imaging)

A total of 197 patients were included in the analyzed studies. 157 patients were subjected to routine re-imaging while the remainder underwent symptom-based imaging (Fig. 7). In the Aldiwani article [33], repeat imaging in the planned group demonstrated two new relevant renal findings in 4% of the patients, namely two PSAs in patients with penetrating renal trauma. Both were grade III injuries and were managed successfully with selective embolization. In the symptoms based imaging group, there were three additional findings of either no relevance to the initial renal injury or not necessitating additional management. Davis et al. demonstrated that among the routine repeat imaging cohort, 31% had a stable injury with 68% displaying resolution on imaging while 20% of patients undergoing clinically indicated re-imaging showed evidence of progression requiring intervention [34]. Addressing the complication of delayed hemorrhage necessitating intervention, four (36%) of 11 patients with clinical evidence of ongoing blood loss, persistent or increasing hematoma, decreasing hematocrit or episodes of hemodynamic instability had injury related complications that were confirmed on repeat imaging including two traumatic PSAs and two AVFs which were all successfully embolized. Conversely, the incidence of such findings in the routine imaging group was zero. While several studies argued against routine re-imaging in stable patients [35–38], Aldiwani [33] advocated for routine repeat imaging in order to avoid the serious consequences of missing such complications. In their series, both findings developed after penetrating trauma. Alternately, clinical indication-based re-imaging did not amount to significant or management altering sequelae. However, given the small number of patients in their subgroup analysis, it’s difficult to stratify indications by mechanism. In contrast to their results are the findings by Davis et al. that negated the need for routine re-imaging. Heterogeneity for this outcome was 71.9% (p = 0.059) [34].

### Grading the evidence

**Fig. 8:**
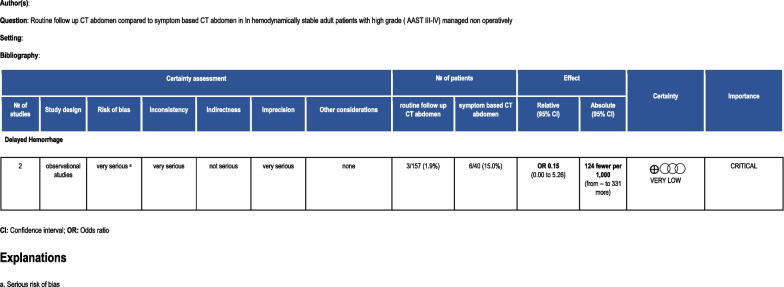
PICO 4 evidence profile

Considering that both studies are single-center, retrospective with relatively small numbers and the confounding risk of publication bias, indirectness, imprecision, inconsistency and population heterogeneity (Fig. 8).

### PICO 4 recommendation

The majority (7) of the workgroup members gave a “no recommendation” vote. In HD stable adult patients with high grade (AAST III–V) renal injuries managed non-operatively (P) no recommendation can be made for or against routine follow up CT abdomen (I) vs. symptom-based CT abdomen (C) to decrease the incidence of delayed hemorrhage necessitating intervention.

#### Summary of recommendation

- PICO 1: In hemodynamically stable adult patients with renal trauma and evidence of active bleeding clinically or radiographically (P), no recommendation can be made for angioemboli-zation (I), versus observation (C) to decrease mortality and risk of surgical morbidity (delayed hemorrhage necessitating intervention and nephrectomy) (O)?
- PICO 2: In hemodynamically unstable patients with a stable zone II hematoma diagnosed intraoperatively (P), we conditionally recommend against renal exploration (I) vs no renal ex-ploration (C) to decrease the incidence of mortality and nephrectomy (O)
- PICO 3: In hemodynamically unstable patients found to have an expanding zone II hematoma necessitating exploration (P), we conditionally recommend against total nephrectomy (I) versus attempted kidney preserving surgery (partial nephrectomy or repair) (C) to decrease mortality, delayed hemorrhage necessitating intervention, AE and need for long term RRT (O).
- PICO 4: In hemodynamically stable adult patients with high grade (AAST III–V) renal inju-ries managed non-operatively (P) no recommendation can be made for routine follow up CT ab-domen (I) vs. symptom based CT abdomen (C) to decrease the incidence of delayed hemor-rhage necessitating intervention.

## Conclusion

### Using these guidelines in clinical practice

In patients who are stable with evidence of retroperitoneal trauma or gross hematuria, axial imaging with intravenous contrast should be obtained. If active extravasation is noted on CT in at least secondary branches of the renal vessels, depending on the patient’s hemodynamics, severity of injury, need for ongoing blood product transfusions, presence of other injuries, availability of in house surgical team, ICU availability, pre-existing renal failure and contrast load on initial scan, patients may undergo angioembolization, or be closely monitored hemodynamically with serial hemoglobin (Hb) assessments and serial creatinine (Cr) and urine output measurements. In the event of deterioration of any of those, repeat imaging and/or angioembolization may be considered.

In case of laparotomy for other indications if a non-expanding hematoma is noted in the retroperitoneum that is not actively being released externally or into the peritoneal cavity through a break of the peritoneal lining, we recommend against renal exploration. In case of hemodynamically unstable patients with obvious enlarging retroperitoneal hematomas suggestive of clinically significant renal bleeding, in which resection is warranted for hemostasis, kidney-preserving surgery is preferrable depending on the patient’s hemodynamics and surgeon’s expertise.

Patients with high grade renal injuries that were managed either operatively or non-operatively should be closely monitored with serial Hb, Cr and urine output in a highly monitored setting. In case of deterioration of either, or even routinely after 48–72 h, repeat contrasted axial imaging may be obtained to determine the presence of delayed hemorrhage necessitating intervention.

## Future directions

All recommendations were supported by very low quality evidence with a predominance of small retrospective studies limited to the English literature. The need for large scale prospective studies addressing these four PICOs is evident. Both the direction and strength of recommendations may change as additional research emerges.

## Supplementary Information

**Additional file 1: **Literature search.

## Data Availability

All data generated or analyzed during this study are included in this published article and its Additional files.
