# Supplementary material for: Management of adult renal trauma: a practice management guideline from the eastern association for the surgery of trauma
Source: BMC Surg. 2023 Jan 27;23:22. doi: 10.1186/s12893-023-01914-x (PMC9881253; doi:10.1186/s12893-023-01914-x)
Supplement: Supplementary file 1 — Additional file 1: Literature search. [file 12893_2023_1914_MOESM1_ESM.docx]

**Additional file 1:**

**Literature search**

(((((((((("Kidney"[mh] OR kidney[tiab] OR renal[tiab]))) AND (("Wounds and Injuries"[Majr:NoExp] OR "Soft Tissue Injuries"[Mesh] OR "Multiple Trauma"[Mesh] OR "Trauma Centers"[mh] OR trauma[tiab] OR traumatic[tiab] OR polytrauma[tiab] OR injury[tiab] OR injuries[tiab] OR injured[tiab] OR injure[tiab] OR injuring[tiab] OR wound[tiab] OR wounds[tiab] OR wounded[tiab]))) AND (("Wounds, Nonpenetrating"[Mesh] OR "Wounds, Penetrating"[Mesh] OR "Contusions"[Mesh] OR "Crush Injuries"[Mesh] OR "Fractures, Bone"[Mesh] OR "Fractures, Cartilage"[Mesh] OR "Lacerations"[Mesh] OR "Hematoma"[Mesh] OR "Hemorrhage"[Mesh] OR "Rupture"[Mesh] OR "Wounds, Gunshot"[Mesh] OR "Wounds, Stab"[Mesh] OR "Burns"[Mesh] OR "Accidental Falls"[Mesh] OR "Accidents, Traffic"[Mesh] OR blunt[tiab] OR crush[tiab] OR crushed[tiab] OR penetrate[tiab] OR penetrates[tiab] OR penetrating[tiab] OR penetrated[tiab] OR penetration[tiab] OR nonpenetrating[tiab] OR "non penetrating"[tiab] OR fracture[tiab] OR fractures[tiab] OR fractured[tiab] OR lacerate[tiab] OR lacerates[tiab] OR lacerated[tiab] OR laceration[tiab] OR lacerations[tiab] OR perforation[tiab] OR perforations[tiab] OR perforate[tiab] OR perforates[tiab] OR perforated[tiab] OR rupture[tiab] OR ruptures[tiab] OR ruptured[tiab] OR falls[tiab] OR falling[tiab] OR abrasion[tiab] OR abrasions[tiab] OR contusion[tiab] OR contusions[tiab] OR "motor vehicle collision"[tiab] OR "motor vehicle collisions"[tiab] OR "motor vehicle accident"[tiab] OR "motor vehicle accidents"[tiab] OR "motor vehicle crash"[tiab] OR "car accident"[tiab] OR "car accidents"[tiab] OR "car crash"[tiab] OR "vehicular accident"[tiab] OR "vehicular accidents"[tiab] OR "vehicular collision"[tiab] OR "vehicular collisions"[tiab] OR "vehicular crash"[tiab] OR "traffic accident"[tiab] OR "traffic accidents"[tiab] OR MVC[tiab] OR "traffic crash"[tiab] OR "automobile crash"[tiab] OR "automobile collision"[tiab] OR "automobile collisions"[tiab] OR "automobile accident"[tiab] OR Hematoma[tiab] OR hematomas[tiab] OR haematoma[tiab] OR haematomas[tiab] OR gunshot[tiab] OR "gun shot"[tiab] OR firearm[tiab] OR firearms[tiab] OR stab[tiab] OR stabbed[tiab] OR burn[tiab] OR burns[tiab] OR burned[tiab] OR Hemorrhage[tiab] OR hemorrhages[tiab] OR hemorrhaged[tiab] OR hemorrhaging[tiab] OR haemorrhage[tiab] OR haemorrhages[tiab] OR haemorrhaged[tiab] OR haemorrhaging[tiab] OR bleeding[tiab] OR bleed[tiab] OR bleeds[tiab] OR bled[tiab] OR "high grade"[tiab] OR "zone 2"[tiab] OR "zone II"[tiab])))) NOT ((Editorial[ptyp] OR Letter[ptyp] OR Case Reports[ptyp] OR Comment[ptyp])))) NOT ((animals[mh] NOT humans[mh])))) AND English[lang
